# Supplementary material for: Identification and Expression Analysis of NAC Gene Family in Weeping Trait of Lagerstroemia indica
Source: Plants (Basel). 2022 Aug 21;11(16):2168. doi: 10.3390/plants11162168 (PMC9413744; doi:10.3390/plants11162168)
Supplement: Supplementary file 1 [file plants-11-02168-s001.zip › Table S1 Specific primers for RT-PCR of LiNAC in L.pdf]

Table S1 Specific primers for RT-PCR of *LiNAC* in *L. indica*

Gene name	Primer sequence (5′-3′)	
LiNAC1	F: CTGGCATCGCACAAGACGGT	R: CCTCCTCCCATTCCTCCTCAA
LiNAC2	F: ATCCCATTTCCGAGGTCGAT	R: TTGCCCGTGGTCTTCCAGT
LiNAC3	F: TGAGACTACGCATCCCGAAAC	R: AGCACCCTCCACTCCACCTT
LiNAC4	F: TCATACTACCGCAACTGGCACC	R: CCATTGAAACCGAACCCATCTC
LiNAC5	F: CCCCATTGCCTAAGGACACG	R: TGGTTGCTGAGGGAGACGGT
LiNAC6	F: AAGCAACCAAGAACTGCCAAGC	R: GCAATCAGGACCCGAACCAC
LiNAC7	F: GCTCACTTCCGTGGGACAATAC	R: CACATCAGGTGCCAACTCTTCTT
LiNAC8	F: ACAAACAAACTCATTAGCGACAGC	R: CTAGGGAGGTGCAAGAACCGT
LiNAC9	F: AACAACAAGAACGATGAGGACAAC	R: CGATTCCCGCCCATGAAA
LiNAC10	F: CTCAACCAACCCCGACAGGA	R: CAGCCCATGAAATCGTAGAAAACA
LiNAC11	F: CTGGGTCCTGTGCCGGATAT	R: CTTCAGTGCTGCTGTTCATGTTG
LiNAC12	F: GAAGTTGCCAGCGGAGTTGT	R: CGGTTCCTGCCCATGAAAT
LiNAC13	F: TTCTCCAACATTTTCTCCCAACAG	R: GCAGGGCTTTCAGCATCTTCT
LiNAC14	F: AGCCCTGGGACTTGCCTGAT	R: TTGGAGCCCGACCTTTGTG
LiNAC15	F: CAGCAATGGAGTTGAAAACGG	R: CCCAATGGCTAAATCTGGAATC
LiNAC16	F: GCGGACCTTTTGGGTTATGC	R: GAAGATTTTGCTGCTGGAGTGG
LiNAC17	F: GAAAGCGGACTCGTTGGGTT	R: CGGTTGAATTATCAAGGGTCTCAT
LiNAC18	F: GCGGACCTTTTGGGTTATGC	R: GAAGATTTTGCTGCTGGAGTGG
LiNAC19	F: CTTGCCAGGTGATTTGAGGG	R: TTCCAGCGACAGGAGCCAT
LiNAC20	F: CTTGCCAGGTGATTTGAGGG	R: TTCCAGCGACAGGAGCCAT
LiNAC21	F: AGAGTCAATGGTTATGGCATCAGG	R: TAGTAGTTGCGGAAAGCAGTCG
