# Supplementary figures and images for: Identification and Expression Analysis of NAC Gene Family in Weeping Trait of Lagerstroemia indica
Source: Plants (Basel). 2022 Aug 21;11(16):2168. doi: 10.3390/plants11162168 (PMC9413744; doi:10.3390/plants11162168)

LiNAC7

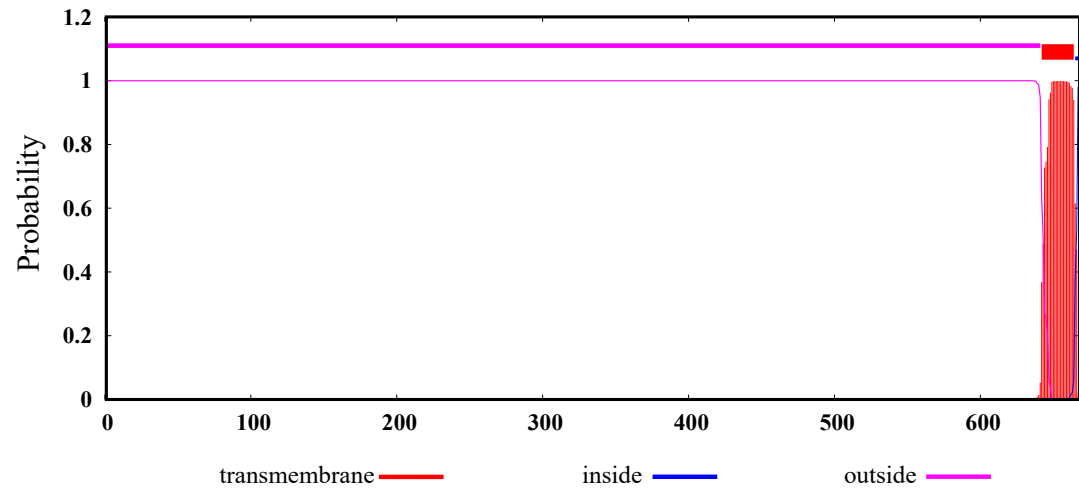

LiNAC16

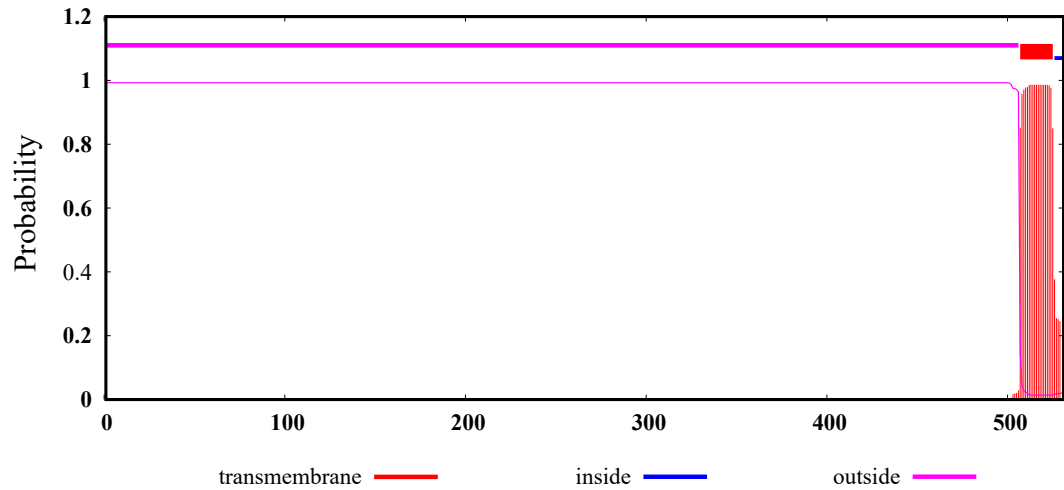

Supplement: Supplementary file 1 [file plants-11-02168-s001.zip › Figure S1. The transmembrane structure of LiNAC7 and LiNAC16.pdf]

Motif 1

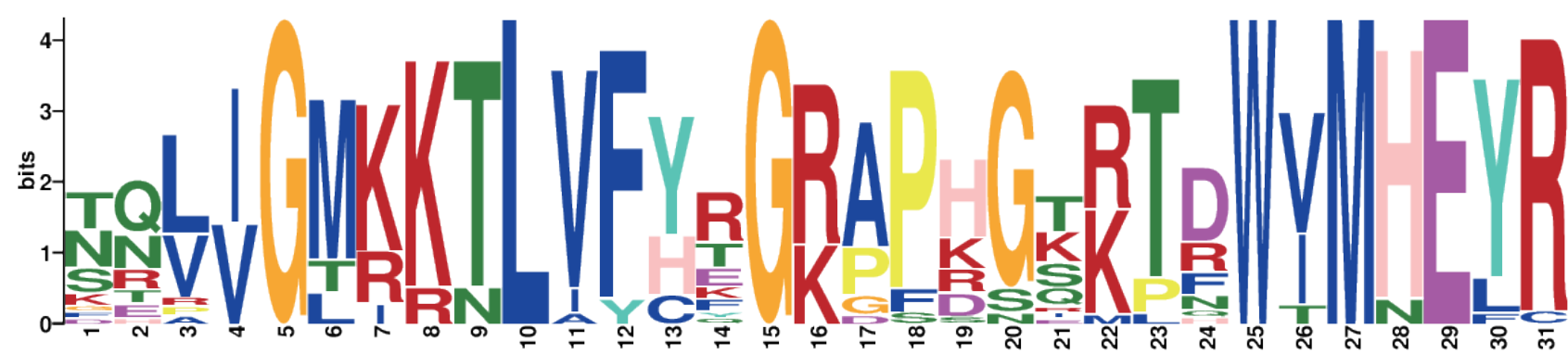

Motif 2

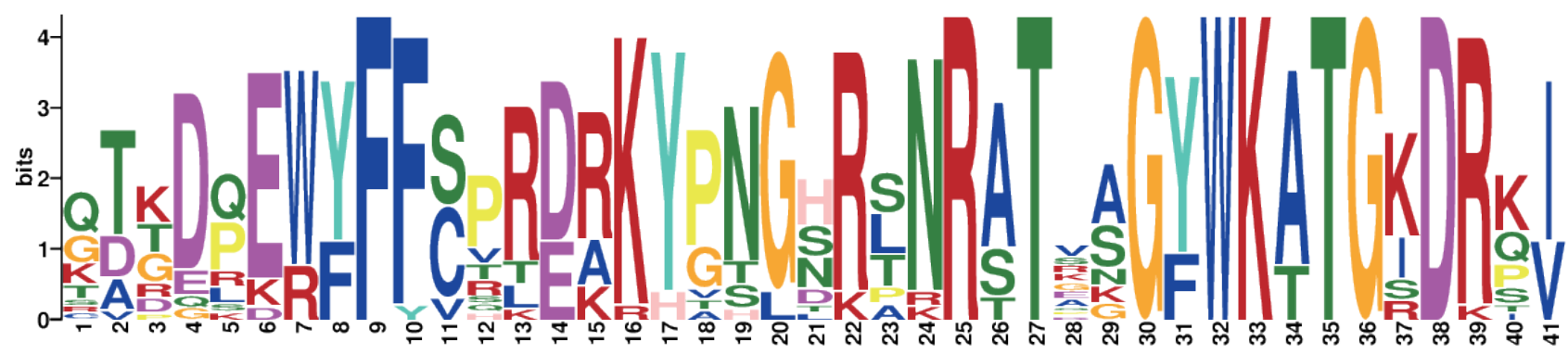

Motif 3

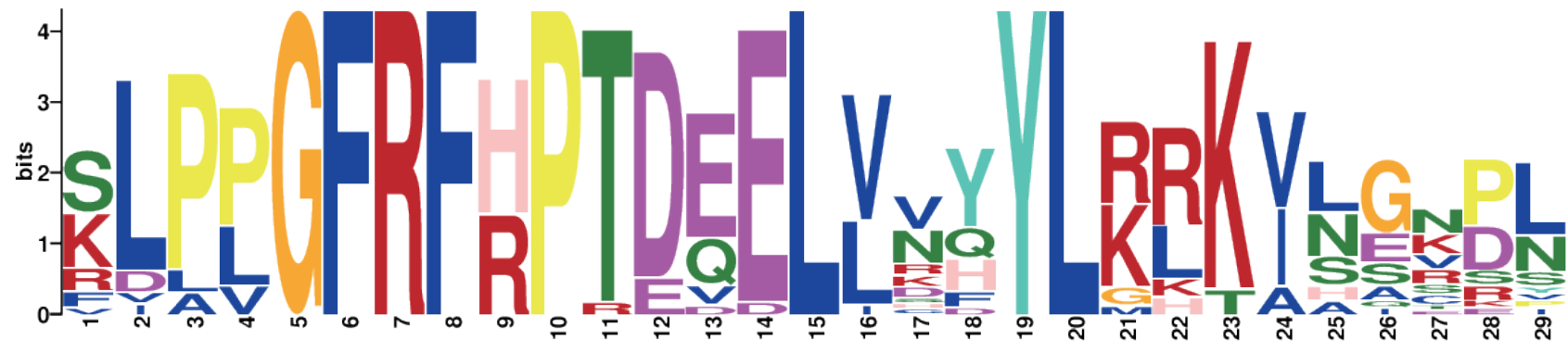

Motif 4

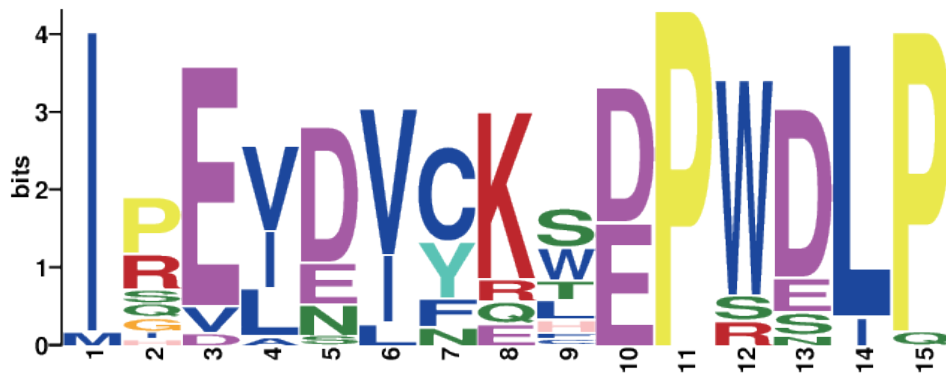

Motif 5

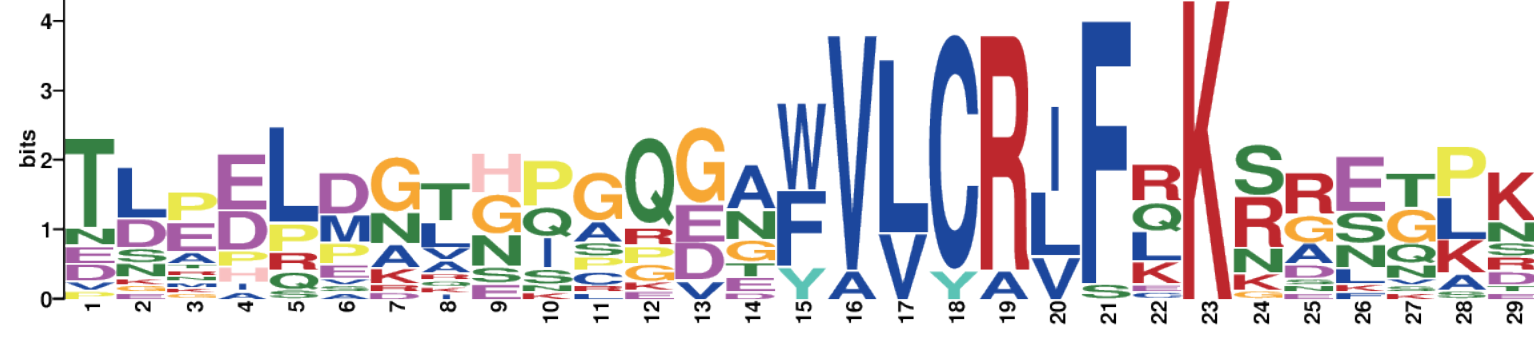

Supplement: Supplementary file 1 [file plants-11-02168-s001.zip › Figure S2. Conserved motifs in five subdomains of LiNACs.pdf]
